# Fall‐applied manure can conserve excess soil‐profile inorganic‐N for the subsequent cropping year

**DOI:** 10.1002/jeq2.70040

**Published:** 2025-05-13

**Authors:** Rodrick D. Lentz, Jim A. Ippolito

**Affiliations:** ^1^ USDA‐ARS Northwest Irrigation and Soils Research Laboratory Kimberly Idaho USA; ^2^ School of Environment and Natural Resources The Ohio State University Columbus Ohio USA

## Abstract

To investigate dairy manure urea fertilizer interactions in cropped soils of the semiarid‐West, we fall‐applied and incorporated (0–0.3 m) soil urea‐N (FertN) rates of 10 (N0), 45 (N1), and 80 mg N kg^−1^ (N2), co‐applied with either no manure or 86 Mg ha^−1^ (dry wt.) stockpiled dairy manure. Soil net N mineralization, inorganic N (InorgN*), and water extractable organic carbon (WEOC*) to a 1.2‐m depth, and silage corn yield and N uptake measurements were used to derive a simple mobile (soluble) N soil budget. The InorgN's descending‐pulse, soil leaching profile contrasted with WEOC's adsorption and complexation profile, in which 95% of the manure‐sourced WEOC accumulated in the 0‐ to 0.6‐m soil layer by summer's end. At the outset, treatments influenced InorgN loading only at the 0‐ to 0.3‐m depth, where doubling FertN from N1 to N2 increased InorgN in non‐manured soils by an average 2.3‐fold, while in manured soils InorgN was unchanged. Manure addition inhibited the availability of the added N2 FertN, possibly by increasing NH_4_^+^ adsorption or its fixation by 2:1 type clay minerals. In response to increasing FertN, net mobile‐N loss from soil profiles between late fall and summer's end: (1) increased from −26.2 to 116 kg ha^−1^ in non‐manured soils and (2) decreased from −54.7 to −338 kg ha^−1^ in manured soils. The one‐time fall manure application stimulated ongoing, variable, and nonsynchronous N‐cycling, which, with recurrent cycling of NH_4_‐N between the soil solution and exchangeable pools, interrupted and delayed transport of excess soil InorgN through soil profiles.

## Core Ideas

- Fall applied manure alters inorganic‐N and water extractable organic carbon (WEOC) (H2O extractable organic C) loads and their transport in soil.
- In the subsequent crop year, these along with induced changes in soil net N mineralization stimulate N‐cycling.
- Despite bigger N and WEOC inputs to manured soil, annual soluble‐N losses were greater in non‐manured profiles.
- N‐cycling changes alone do not seem to fully explain this result.
- NH4‐N cycling among soluble, absorbed, and fixed soil pools may inhibit nitrification and soluble‐N losses.

**Abbreviations**
InorgN: soil inorganic N
minN: net N mineralized
WEOC: water extractable organic C
WEON: water extractable organic N

## 1 INTRODUCTION

Past research conducted in irrigated soils has shown that one‐time, fall‐applied manure treatments increased the 3‐year, cumulative, net N mineralized (minN) in the 0‐ to 0.3‐m depth by an average of 1.85x relative to soils with no manure, either with or without an inorganic‐N fertilizer treatment (Lentz et al., 2011). The impact of heavier manure treatments (45–67 Mg ha^−1^ dry wt.) was strongest, particularly at the 0.3‐ to 0.6‐m depth where these rates produced an average 5.5‐fold greater 3‐year cumulative net N mineralization than the 0–23 Mg ha^−1^ dry wt. treatment group (Lentz et al., 2011). Furthermore, the magnitudes of temporal net‐N‐mineralization values for individual seasonal periods were generally more extreme at 0.3‐ to 0.6‐m depths than at 0‐ to 0.3‐m, that is, the 0.3‐ to 0.6‐m soil zone produced greater positive net‐N‐mineralization values and more negative net‐N‐mineralization values (greater N immobilization) than the 0‐ to 0.3‐m zone (Lentz et al., 2011). Lentz et al. (2011) reported that a substantial portion of N cycling in the soil occurred in the 0.3‐ to 0.6‐m zone, where N mineralization and immobilization are likely facilitated by soluble materials leached from the soil above to provide a C or N source that stimulates microbial activity (He et al., 2009). Thus, if 0.3‐ to 0.6‐m deep soils contain available inorganic N (InorgN) but are starved of C, an influx of soluble organic C from manure can instigate a cycle of intense N immobilization followed by N mineralization (Lentz et al., 2011). Evidence confirming intense soil N cycling was reported by Lentz and Lehrsch (2018); their nitrate stable isotope (^15^N‐NO_3_ and ^18^O‐NO_3_) study found that the isotopic signature of fertilizer nitrate was rapidly transformed through a repeated series of immobilization–mineralization cycles as it transited the soil profile (0‐ to 1.2‐m depth) in percolating water.

Other researchers have reported that manure had inconsistent effects on soil N immobilization and N leaching (Fan et al., 2017; Malcom et al., 2019; Nishio et al., 2001; Tarkalson et al., 2006). Manure may influence soil nitrogen transformations and movement by increasing soil organic matter content. Soil organic matter and the “clay‐humic complex” influences adsorption of dissolved and exchangeable NH_4_‐N (Al‐Saedi et al., 2021; Boatman & Murry, 1982; Rosenfeld, 1979), particularly in calcareous soils (Al‐Saedi et al., 2021).

Based on our past research, we hypothesized that the application of manure and attendant leaching of its soluble organic materials would result in intensive N‐cycling, which slows N translocation and loss through the profile and root zone. In effect, repeated, successive N‐cycling episodes would act like a dynamic storage repository for InorgN in the soil profile; dynamic in the sense that the InorgN storage is temporary but with the potential to slow InorgN N leaching losses. The objective of this experiment was to determine the effect of a fall manure application on net N mineralization and leaching of InorgN and water extractable organic carbon (WEOC) in soils containing differing InorgN contents. Measured parameters were monitored through the end of the subsequent cropping season.

**Core Ideas**

- Fall applied manure alters inorganic‐N and water extractable organic carbon (WEOC) (H2O extractable organic C) loads and their transport in soil.
- In the subsequent crop year, these along with induced changes in soil net N mineralization stimulate N‐cycling.
- Despite bigger N and WEOC inputs to manured soil, annual soluble‐N losses were greater in non‐manured profiles.
- N‐cycling changes alone do not seem to fully explain this result.
- NH4‐N cycling among soluble, absorbed, and fixed soil pools may inhibit nitrification and soluble‐N losses.

## 2 MATERIALS AND METHODS

### 2.1 Experimental design

The experimental design was a randomized complete block with four replicates and six soil treatments applied in late fall prior to the growing season (Treatments 1–6; Table 1). The six treatments included two levels of manure amendment (no manure: Treatments 1–3, or late fall manure: Treatments 4–6) and three levels of total inorganic N fertilizer (FertN) applied in late fall (i.e., FertN0 = 10 mg N kg^−1^, FertN1 = 45 mg N kg^−1^, and FertN2 = 80 mg N kg^−1^ soil for Treatments 1–3 and 4–6, respectively). No additional pre‐ or post‐plant fertilizer was applied to plot soils. The FertN0 treatment represented essentially an unfertilized, late‐fall soil with minimal total soil inorganic‐N content and hence the control N level. Manure was applied first followed by FertN, and then both materials were incorporated into the 0‐ to 0.3‐m soil layer. The N1 rate (45 N kg^−1^) can yield 14.5 Mg ha^−1^ silage (dry wt.) and N2 can yield 29 Mg ha^−1^ silage (Brown et al., 2010). The experiment was repeated (referenced as Experiment 2) in the following year on new plots created in an immediately adjacent field. In Experiment 2, we applied roughly 2.4x more manure to plots than in Experiment 1. Field manure rates used by local producers can vary widely and we wanted experimental results to capture this range. We established the plots in the fall 2014 for Experiment 1 (2015 crop year) and measured net N mineralization in all plots using buried bags at 0‐ to 0.3‐m and 0.3‐ to 0.6‐m depths during the subsequent winter, spring, early summer, and late summer periods. Incremental soil sampling to 1.2‐m depth (Section 2.4) determined InorgN and WEOC in (i) early fall 2014 (baseline conditions), (ii) late fall 2014 after manure and fertilizer additions, (iii) spring of the 2015 crop year, (iv), and (v) summer's end 2015. We planted corn (*Zea mays* L.) in plots and measured corn silage yield and total C and N in harvested biomass in fall 2015 and 2016. Table S1 lists specific dates for each sampling event. In Experiment 2, a similar approach was followed from fall 2015 through summer's end 2016.

**TABLE 1:** Application rates (all on a dry wt. basis) for manure and fertilizer, and manure total element concentrations, in each year of the study.

Crop year	Stockpiled dairy manure					Urea applied to obtain soil inorganic N target		
	Concentration		Bulk application rate			Treatments		
	C (g kg^−1^)	N (g kg^−1^)	Solids (Mg ha^−1^)	C (Mg ha^−1^)	N (Mg ha^−1^)	1,4 (kg ha^−1^)	2,5 (kg ha^−1^)	3,6 (kg ha^−1^)
2015	249	16.1	50.6	12.6	0.815	0	156	312
2016	232	16.3	121	27.9	1.960	0	168	325

*Note*: Treatments 1, 2, and 3 received no manure while 4, 5, and 6 received manure. The targeted total inorganic N concentrations in the 0‐ to 0.3‐cm soil layer were 10, 45, and 80 mg N/kg for Treatments 1 and 4, Treatments 2 and 5, and Treatments 3 and 6, respectively.

Abbreviation: N, total N.

### 2.2 Site, soils, and amendments

We conducted the experiment on sprinkler irrigated Portneuf silt loam (coarse‐silty, mixed superactive, mesic Durinodic Xeric Haplocalcids) with 1.4% slopes near Kimberly, ID (42 E 31′ N, 114 E 22′ W, elevation of 1190 m). The 0–0.3 m soil contained 200 g kg^−1^ clay, 560 g kg^−1^ silt, 17 g kg^−1^ total C, 7.6 g kg^−1^ inorganic C, 9.4 g kg^−1^ total organic carbon (TOC), 0.94 g kg^−1^ total N, exhibited electrical conductivity (EC) of 0.04 S m^−1^, exchangeable sodium percentage of 1.5%, pH of 7.6, and a cation exchange capacity (NH_4_OAc @ pH 7) of 19 cmol_c_ kg^−1^. Dominant soil clay minerals include illite > > kaolinite = montmorillonite > vermiculite (Lewis, 1986). Soil particle size analysis was determined using the hydrometer method, applied after removal of organic matter. Soil total C and total N were determined on a freeze‐dried sample with a Thermo‐Finnigan FlashEA1112 CN analyzer (CE Elantech Inc.), total inorganic C using a pressure‐calcimeter (Sherrod et al., 2002), and TOC by difference. The soil EC and pH were determined on a saturated paste extract.

A spring barley crop (*Hordeum vulgare* L.) grown on plot soils in the year preceding each experiment effectively reduced InorgN concentrations in initial profile soils to < 10 mg kg^−1^. No manure had been applied to the soils since 1986. The applied solid dairy cattle manure (*Bos* species) had been stockpiled at a local dairy during the summer months and thus contained minimal concentrations of bedding straw. Amendments were applied in a manner identical to Lentz et al. (2012). Manure rates were chosen to approximate the heavier rates used in a previous study (Lentz et al., 2011). Manure total C and total N were determined on a freeze‐dried sample with a Thermo‐Finnigan FlashEA1112 CN analyzer (CE Elantech Inc.).

### 2.3 Field operations

The sequence of experimental field procedures employed is given in Table S1. Preplant sampling verified that soil P and K availability was adequate for the crops (Brown et al., 2010). We applied manure to designated plots using a small plot manure spreader, applied urea fertilizer with hand‐held spreader, and incorporated both to a depth of 0.13 m with a disk plow. In spring, the plots were roller harrowed and planted to silage corn (P9188AM, Pioneer) in 0.75‐m rows at 0.15‐m plant spacing, with an approximate density of 8.6 × 10^4^ seeds ha^−1^. The crop received two generic glyphosate (N‐[phosphonomethyl] glycine) herbicide applications at labeled rates in June or early July. A solid set sprinkler system supplied water to the crops as required to meet evapotranspiration demand, typically in 12‐h sets.

### 2.4 Soil profile sampling and analyses

Three separate 5.7‐cm diameter soil cores were collected from the 0‐ to 0.6‐m depth, with each core divided into 0‐ to 0.15‐, 0.15‐ to 0.3‐, and 0.3‐ to 0.6‐m depths. Similar depths within each plot were composited. Two additional cores were collected from the 0.6‐ to 1.2‐m depth, with each core divided into 0.6‐ to 0.9‐ and 0.9‐ to 1.2‐m depths, with similar depths from each plot also composited. Soil from each depth was passed through a 4‐mm screen, mixed, 25% was retained in its moist state for water content and WEOC determinations, and the remaining was dried at 35°C and crushed to pass a 2‐mm screen.

Soil profile InorgN in dried soil samples was extracted using a 2 M KCl solution (Mulvaney, 1996). The NO_3_‐N concentration in each extract was determined within 6 h of extraction using an automated flow injection analyzer (Lachat Instruments) after cadmium reduction (Method 12‐107‐04‐1‐B) while NH_4_‐N concentration was determined simultaneously using a salicylate‐hypochlorite method (Method 12‐107‐06‐2‐A). The InorgN concentration, per soil layer, equaled the sum of NO_3_‐N + NH_4_‐N (mg N kg^−1^ soil). The InorgN mass equaled the product of InorgN concentration and mean bulk density of the soil layer (kg ha^−1^). The soil profile InorgN mass (InOrgN*) was the sum of InorgN mass across all five soil depths (i.e., 0–1.2 m).

Soil profile WEOC was determined on moist soil profile samples. Fifty mL of reverse osmosis (RO) water was mixed with 12.5 g soil (corrected for water content) on a box shaker (60 cycles min^−1^) for 30 min at room temperature, then processed in a refrigerated centrifuge at 20,000 × *g* for 20 min to remove >30‐µm‐sized particles and sediment. The clear supernatant was stabilized with a saturated boric acid (H_3_BO_3_) solution (1 mL per 100 mL sample) and stored at 4°C until analyzed for WEOC as non‐purgeable organic carbon using a Shimadzu TOC‐5050A instrument (Shimadzu Scientific Instruments). The WEOC concentration, WEOC mass, and WEOC* are defined in a similar fashion as InorgN above.

### 2.5 Soil profile net N mineralization

Soil N mineralization is strongly and positively related to extractable soil organic matter (Ros et al., 2011). Since this organic matter occurs primarily in the 0‐ to 0.6‐m soil depth, the net N mineralization in the 0‐ to 0.6‐m depth presumably provided a reasonable estimate of soil profile net N mineralization (0‐ to 1.2‐m soil depth). We measured net N mineralization in plot soils using the buried bag method (Lentz & Lehrsch, 2012; Lentz et al., 2011; Westermann & Crothers, 1980). In early winter, plot soils from 0‐ to 0.3‐m and 0.3‐ to 0.6‐m depths were collected separately and one bag per depth per plot prepared and installed (Table S1). Again, in spring, plot soils from the two depths were collected and three bags depth^−1^ plot^−1^ prepared and installed (Table S1). We measured InorgN and soil water content of bag soils prior to installation and again after bags were retrieved (Table S1). The net N mineralization during the period between bag burial and retrieval was calculated as the difference (retrieved minus initial) between the two soils’ InorgN concentrations. A positive difference indicated net N mineralization, while a negative value indicated net N immobilization during the period. If the water contents of the initial and retrieved bag markedly differed, the bag's result was scrutinized for accuracy. The minN measured per soil layer was reported directly as mg N kg^−1^ soil. We calculated the cumulative net N mineralization per soil layer (CumMinN) by converting minN to units of kg ha^−1^ and summing across the four periods from winter through late summer. CumMinN* is the net N mineralization in the 0‐ to 1.2‐m soil profile from winter to late summer. Details of the field and laboratory procedures are reported by Lentz et al. (2014).

### 2.6 Plant sampling and analyses

Corn silage yields were measured by hand clipping corn plants (3 cm above soil surface) from 3‐m lengths of two adjacent rows within each plot. The sample was weighed and then chopped, and a subsample was collected, dried at 65°C, and ground in a Thomas Wiley mill to pass an 865‐µm screen. We determined corn biomass total N concentration by combustion using a Thermo‐Finnigan FlashEA1112 CN analyzer (Thermo‐Finnigan). Harvested biomass N (kg N ha^−1^) was computed as the product of corn silage yield (dry wt.) and mean biomass N concentration.

### 2.7 Calculations and statistical analysis

We constructed a simple mobile N budget for soil profiles in each plot and year, which tracked soluble N inputs and outputs in the 0‐ to 1.2‐m soil profile, except leaching losses. The Net mobile N loss (kg ha^−1^) within the soil profile from late fall through summer's end was computed as the quantity (*A* − *B*), where *A* is late‐fall soluble profile N + input mobile N and *B* is summer‐ending soluble profile N + output mobile N. Hence, for the given sampling time:

$$A={\mathrm{WEON}}^{*}+\;\;{\mathrm{InorgN}}^{*}+\;\;{\mathrm{CumMinN}}^{*}$$

$$\begin{array}{ccc} B & = & {\mathrm{WEON}}^{*}+\;\;{\mathrm{InorgN}}^{*}+\;\;\mathrm{harvested}\;\mathrm{biomass}\;N\\ & & +\;\;\mathrm{residual}\;\mathrm{biomass}\;N+N\;\mathrm{lost}\;\mathrm{in}\;\mathrm{gas}\;\mathrm{emissions} \end{array}$$

The “*” postscript denotes the quantity present in the 0‐ to 1.2‐m soil profile. All values are given as kg ha^−1^. The organic N in soil WEOC (WEON [water extractable organic N]) was estimated as the product of measured WEOC and the mass ratio (N in soil WEOC/soil WEOC) reported by Embacher et al. (2008). The N in residual biomass (unharvested corn crowns and roots) was calculated from measured harvested biomass N (current study) multiplied by the mass ratio (residual biomass‐N/harvested biomass‐N), where the ratio was determined previously for corn crops in a neighboring field (Lentz et al., 2014). The N_2_O emissions from our plot soils were approximated using neighboring field measurements reported by Lentz et al. (2014). Since we did not measure leaching losses from experimental plots, we assumed that a positive Net mobile N loss value indicated a net soluble‐N leaching loss from soils, whereas a negative value indicated that most mobile N was immobilized in microbial biomass or adsorbed or precipitated in other soil organic solids.

The displayed mean values and standard errors of the mean for soil InorgN and WEOC at each soil depth and mobile N budget components were calculated using PROC Means in SAS version 9.2 (SAS Institute Inc., 2012). The net N‐mineralization, corn silage yield, and mobile‐N budget data were examined via analysis of variance (ANOVA) using PROC Mixed in SAS (SAS Institute Inc., 2012). The statistical model included treatment and year as the fixed effect and block and treatment × block as the random effect and included the four orthogonal class comparisons (no‐manure vs. manure, no‐FertN vs. FertN, and linearity of fertilizer level with or without manure).

## 3 RESULTS AND DISCUSSION

Climate and soil temperature conditions present during the study are discussed in Supporting Information data.

### 3.1 Soil InorgN concentrations and loads

**FIGURE 1:**
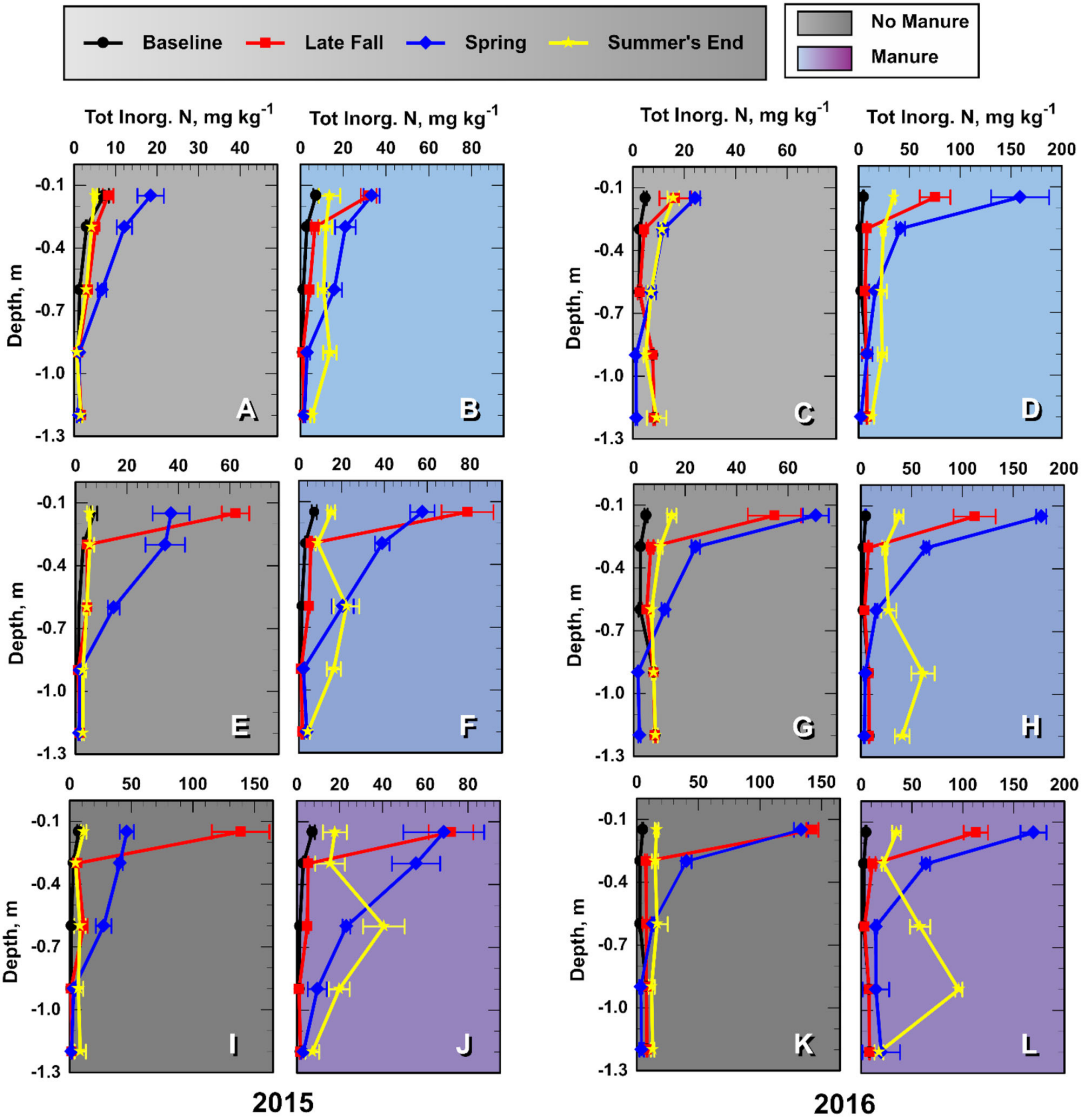
Mean soil profile inorganic‐N (InorgN) concentrations (mg kg^−1^) in the soil layer immediately above the noted soil depth increment at four consecutive sampling times: baseline, late fall after treatment, then spring and late summer during the growing season. Light, medium, and dark shades indicate N0, N1, and N2 fall inorganic‐N treatments, respectively. Each leg of the error bars represents one standard error of the mean (*n* = 4).

**FIGURE 2:**
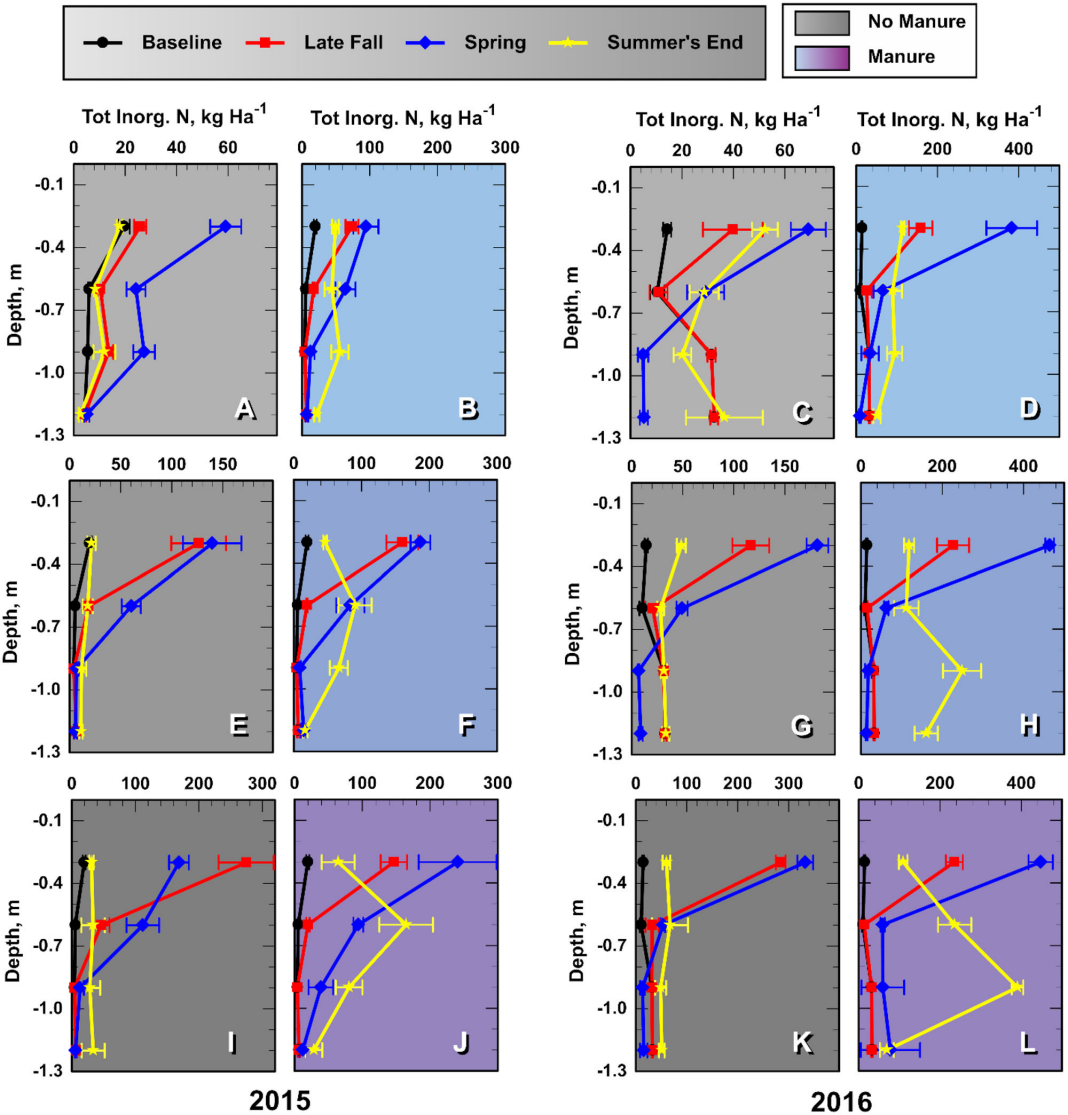
Mean soil profile inorganic‐N (InorgN) loading (kg ha^−1^) in the 0.3‐m soil layer immediately above the noted soil depth increment at four consecutive sampling times: baseline, late fall after treatment, then spring and late summer during the growing season. Light, medium, and dark shades indicate N0, N1, and N2 fall inorganic‐N treatments, respectively. Each leg of the error bars represents one standard error of the mean (*n* = 4).

Mean soil profile inorganic‐N (in concentration and kg ha^−1^) are presented in Figures 1 and 2. Note that each symbol in the graphs indicates the mean value for the soil layer immediately above the depth increment present on the *y*‐axis. The profile configuration of soil InorgN at the initial baseline sampling was relatively similar among all plots but differed as a function treatment and year at other sampling times (Figure 1). In both non‐manure and manure plots, the InorgN concentrations near the soil surface spiked after the fertilizer application and then gradually increased at lower depths as soluble N leached downward in the profile. In 2016 particularly, the surface soil InorgN concentrations were greater in spring relative to the previous late fall, which we attributed to the accumulation of mineralized N (Figure 1A,C,D,G,H,L). In 2016, a greater manure application and perhaps a warmer early spring relative to 2015 (Figure S1), likely contributed to the greater winter‐to‐spring InorgN increase in Experiment 2 (e.g., Figure 1A,B vs. 1C,D) (Lentz et al., 2011; Lentz, unpublished data, 2025). It was apparent that Inorg N concentrations leached more rapidly through non‐manured soil profiles than in manured since, by late summer, the InorgN pulse had disappeared from non‐manured soils but was still present in manured plots (Figure 1). This manure‐induced delay in InorgN leaching was also observed by Malcom et al. (2019).

Results of the ANOVAs investigating soil profile InorgN* treatment responses for late fall, spring, and late summer sampling times are presented in Table S2. Both FertN and manure increased soil profile InorgN loads, except at the late fall sampling date (Table S2). In late fall, the overall influence of manure on InorgN loading was confounded by a FertN‐manure interaction, that is, the positive effect of manure addition on the soil InorgN loading decreased as FertN increased from N0 to N2 (Table S2). Note that treatment effects on InorgN loading below 0.3 m were minor at the late fall sampling, so surface soil impacts dominated the results (Figure 2). When we examined the 0–0.3 m soil in late fall, we found that the average (Experiments 1 and 2) manure InorgN‐loading ratio (no manure/manure) declined from 3.5 for N0 soil (116/32.8 kg ha^−1^), to 1.6 for N1 soil, and to 0.7 for N2 soil (Table S3). In Experiment 1 (2015) 0–0.3 m, late‐fall sampled soils, the effect was even more pronounced; the manure InorgN‐loading ratio for the N2 soil was 0.5 (Figure 2I,J). These results suggest that in late fall, FertN application inhibited soil InorgN loading in manure soils relative to non‐manured. This topic is further discussed in Section 3.5.

### 3.2 Soil WEOC concentrations and loads

**FIGURE 3:**
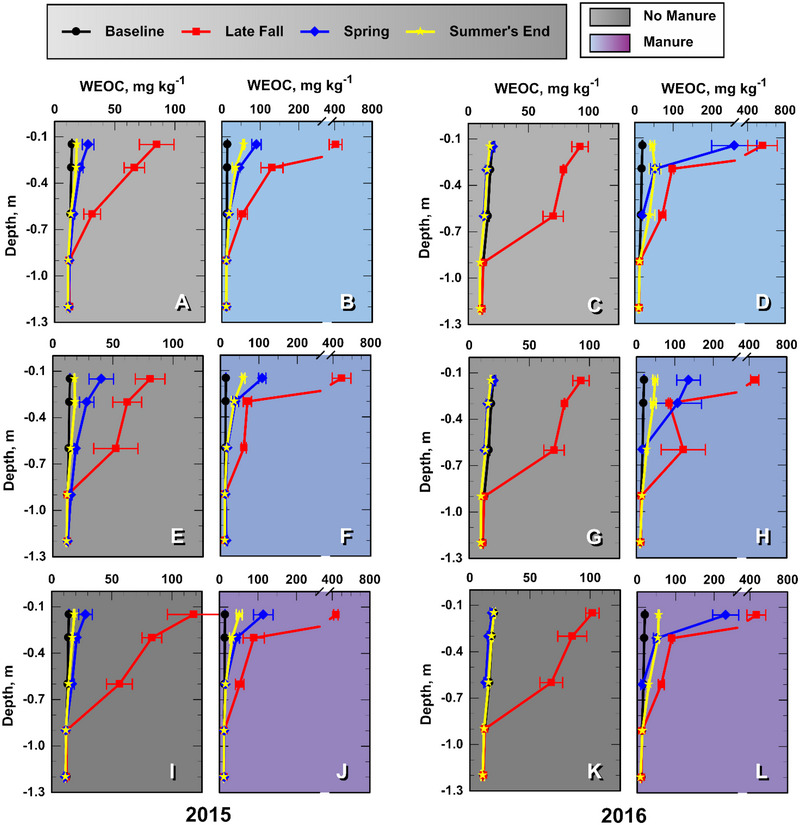
Mean soil profile water extractable organic carbon (WEOC) concentrations (mg kg^−1^) in the soil layer immediately above the noted soil depth increment at four consecutive sampling times: baseline, late fall after treatment, then spring and late summer during the growing season. Light, medium, and dark shades indicate N0, N1, and N2 fall inorganic‐N treatments, respectively. Each leg of the error bars represents one standard error of the mean (*n* = 4).

**FIGURE 4:**
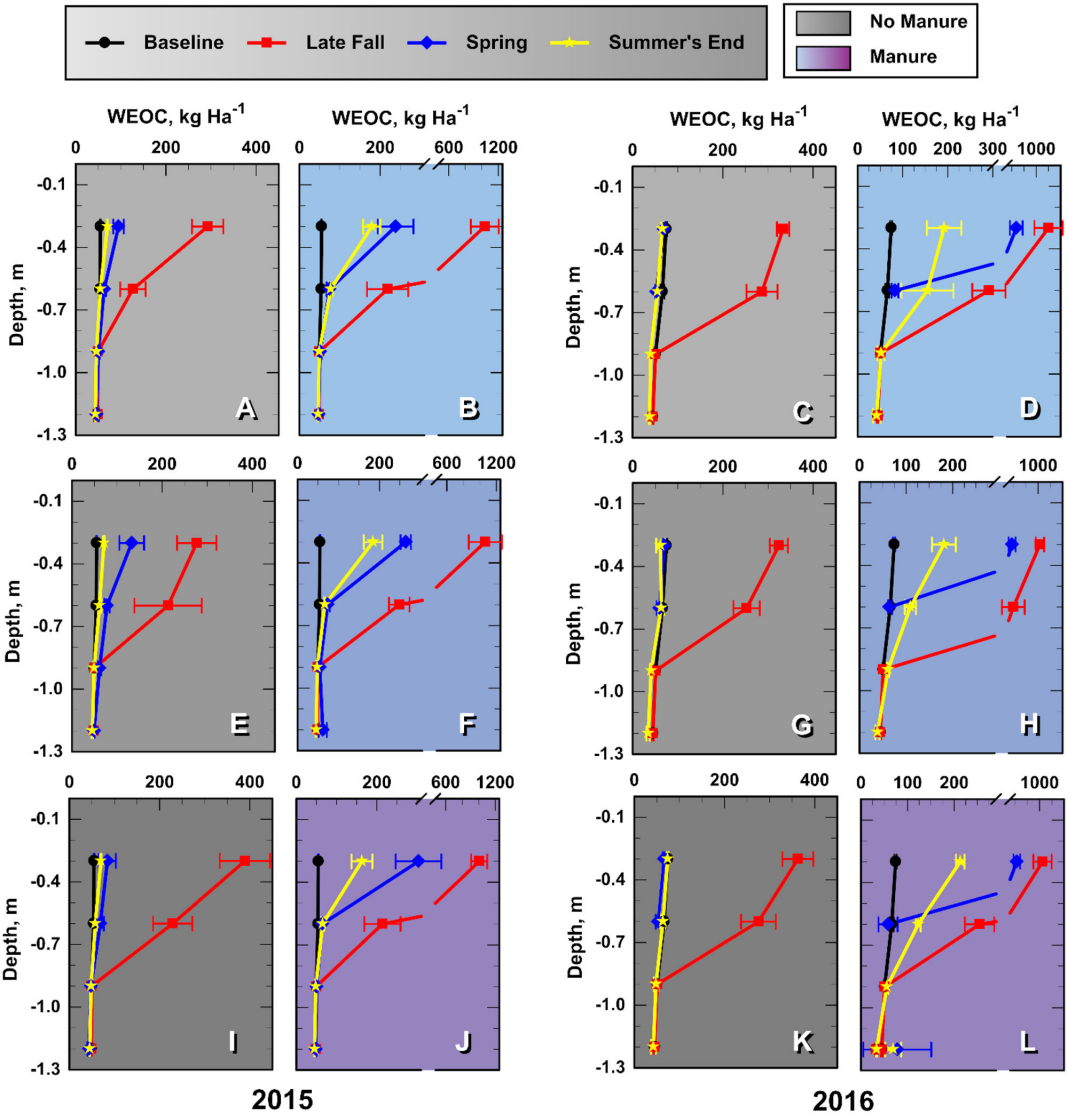
Mean soil profile water extractable organic carbon (WEOC) loading (kg ha^−1^) in the 0.3‐m soil layer immediately above the noted soil depth increment at four consecutive sampling times: baseline, late fall after treatment, then spring and late summer during the growing season. Light, medium, and dark shades indicate N0, N1, and N2 fall inorganic‐N treatments, respectively. Each leg of the error bars represents one standard error of the mean (*n* = 4).

Mean soil profile WEOC (in concentrations and kg ha^−1^) are presented in Figures 3 and 4. Note that each symbol in the graphs indicates the mean value for the soil layer immediately above the depth increment present on the *y*‐axis. The WEOC concentrations in the late fall soil profile increased relative to baseline soil samples collected 2–4 weeks prior, regardless of manure treatment (Figure 3). This was likely due to plant residue (spring barley) decomposition processes that prevailed at this time and was likely amplified by the nearly 2‐week dry period that preceded soil tillage and the late fall soil sampling (Angers et al., 2006; De Mastro et al., 2019; McCarty & Bremner, 1992).

Manure treatments as a class increased mean late‐fall WEOC loading fivefold (*p *< 0.0001) in the 0–0.15 m soil and 1.2‐fold (*p *< 0.04) in the 0.15–0.3 m soil relative to non‐manured soils, but had little influence on soil WEOC at depths below 0.3 m (*p *> 0.22; Table S4). Similar observations were reported by Chantigny (2003), Angers et al. (2006), and Embacher et al. (2008). By summer's end, 95% of the manure‐sourced soil‐WEOC load remained in the 0‐ to 0.6‐m layer (Table S4).

The soil WEOC loading profiles differed from those of InorgN as they did not depict a well‐defined WEOC pulse that descended deeper with time (Figure 4). This results because mineralization, adsorption, and complexation processes limit soluble organic matter transport through the soil profile (Embacher et al., 2008; Hassouna et al., 2010; Kalbitz et al., 2000; Long et al., 2015). The observed attenuation of soil WEOC loads at the 0.6‐m depth at summer's end suggests that much of the mobile WEOC is retained or mineralized within the rooting zone. Even though manure increased soil profile (0–1.2 m) WEOC load (WEOC*) an average 2.5‐fold over that of non‐manured soils in late fall (1364 vs. 557 kg ha^−1^; *p *< 0.0001; Table S4), apparently little of the added WEOC was transported below 0.9‐m depth (Figure 4). It is possible that some WEOC was altered at deeper depths, rendering it insoluble (Hassouna et al., 2010). Leaching losses of dissolved organic carbon from manured soils at 1.2‐m depths are reported to be relatively small: 5.5–6.7 kg ha^−1^ year^−1^ under rainfall and 10 kg ha^−1^ year^−1^ under furrow irrigation (Lentz et al., 2014; Long et al., 2015). Finally, our experimental results indicate that FertN treatments had no influence on WEOC loading profiles (Table S4).

### 3.3 Net N mineralization

Overall, the manure effect on CumMinN was more dominant (*p *< 0.01) than that of FertN treatments (*p *> 0.23) (Table 2). Relative to non‐manured plots, manure treatments increased CumMinN for 0‐ to 0.3‐m and 0.3‐ to 0.6‐m depths, and as a result increased CumMinN* an average 1.5‐fold. As a class, FertN treatment weakly influenced CumMinN or CumMinN* (Table 2). This result contrasts with other research that showed fertilizer N additions either substantially suppressed, or increased, N mineralization (Carpenter‐Boggs et al., 2000; Kalala et al., 2022). When we analyzed minN results on an individual period and year basis, we found that (i) manure impacted minN primarily during the spring and early summer periods in the 0‐ to 0.3‐m soil depth, (ii) the effect of FertN on minN generally was nonlinear for non‐manured soils as well as manured soils (Table S5; Figures S3 and S4), and (iii) in some cases, manure produced opposing effects on minN. For example, at 0‐ to 0.3‐m soil depth during the spring, manure increased net N mineralization in 2015 (*p *< 0.0001) but decreased it in spring 2016 (*p *< 0.016) (Figure S3). Differences in meteorological or environmental conditions between years may have caused N mineralization to dominate in spring 2015 and immobilization to dominate in spring 2016 (Figures S1 and S3). The presence of manure, particularly in higher amounts stimulate both processes (Lentz et al., 2011), leading to greater minN in spring‐2015 and more negative minN in spring‐2016, compared to no manure (Figure S3).

**TABLE 2:** Effect of FertN and manure treatments on cumulative net N mineralization from late fall through summer's end for individual soil depths (CumMinN) and summed over both depths, as an estimate for the 0‐ to 1.2‐m profile (CumMinN*), including class comparisons (contrasts) (*n* = 4).

	CumMinN				CumMinN*	
	0–0.3 m		0.3–0.6 m		0–1.2 m estimate	
Source of variation	*p* values					
Treatment	ns		ns		ns	
Year	ns		*		**	
Treatment × year	ns		**		ns	
Contrasts						
No‐Man vs. Man	*		*		**	
No‐FertN vs. FertN	ns		ns		ns	
FertN linear (No‐Man)	ns		ns		ns	
FertN linear (Man)	ns		ns		ns	
Factor (kg ha^−1^)						
Treatment	2015	2016	2015	2016	2015	2016
1	104	197	26.2	30.9ab	130	228
2	110	113	35.9	42.0ab	146	155
3	115	213	60.0	10.3b	174	223
4	206	216	13.2	85.3ab	220	302
5	210	137	27.2	90.5a	238	227
6	179	240	56.2	80.6ab	235	320
Class comparisons						
No‐manure	109b	175	40.6	27.1b	150b	202b
Manure	199a	198	32.2	85.4a	231a	283a

*Note*: For each treatment or class within year, means followed by the same letter are not significantly different. Letters are not displayed if the effect was not significant in the ANOVA.

Abbreviations: FertN, organic‐N added; FertN linear, inorganic N levels are linearly related; Man, manure; No‐FertN, no inorganic‐N added; No‐Man, no‐manure; ns, not significant.

* and ** denote significance at 0.05 and 0.01 probability levels, respectively.

Increasing manure application for Experiment 2 relative to Experiment 1 increased the range of manure minN values produced across measurement intervals, with greater maxima and lesser minima resulting for Experiment 2 at both soil depths, but particularly the 0‐ to 0.3‐m depth (Figures S3 and S4). A similar response of increasing manure application on period net N mineralization was observed by Lentz et al. (2011), however, the current study saw period net N mineralization values oscillate most strongly at 0‐ to 0.3‐m depth, whereas Lentz et al. (2011) observed the stronger oscillation at 0.3‐ to 0.6‐m depth (Figures S3 and S4). The oscillation in net mineralization indicates that during some seasonal periods, manured soils experience widely disproportionate mineralization and immobilization rates, leading to large differences in net‐N‐mineralization output during the growing season. Additionally, net‐N‐mineralization values among replicates exhibited large variability, such that the median coefficient of variation (CV) across all experimental units at each depth, period, and year was 48%. Median CVs for no‐manure plots generally increased with FertN (29%, 44%, and 53%) while median CVs for manure plots were constant as FertN increased (55%, 49%, and 50%), hence manure generally increased the CV of net‐N‐mineralization values relative to no‐manure plots. This variation would be expected if immobilization–mineralization cycling rates varied in space and were nonsynchronous in time within the soil. Gross mineralization rates tend to be more consistent across arable soils, suggesting that the phasing of immobilization cycles varied most in time and space (Murphy et al., 2000). We conclude that manure decisively stimulated N‐cycling in these soil profiles and the resulting progression of immobilization‐mineralization sequences occurred in a largely nonsynchronous and variable manner throughout the following year. This suggests that manure‐induced N‐cycling is dynamic and ongoing and its continual processing of soil InorgN acts to interrupt and delay InorgN transport through the soil profile.

Finally, the mean CumMinN produced in the year after manure application was influenced by manure and soil depth (*p *< 0.02), but the manure × depth interaction was relatively weak (*p *= 0.26; Table S6). Thus, the proportion of CumMinN produced at the 0.3‐ to 0.6‐m depth relative to that of 0‐ to 0.6‐m depth was similar for no‐manure (19.4%) and manure (23%) (Table S6). These proportions were like values reported by Lentz et al. (2011) for a similar soil, manure application, and incubation period: 24% for no‐manure and 27% for manure.

### 3.4 Silage yield and N‐uptake in crop biomass

Silage yields increased with increasing FertN but generally were not influenced by manure application (Table 3). Silage yields decreased with year and the yield response pattern also differed by year (*p *< 0.03) (Table 3). The 2016 crop yield was 39% less than that of 2015, which we attributed primarily to cooler summer air temperatures in 2016 combined with heavy weed pressure from an infestation of field bindweed (*Convolvulus arvensis* L.). In 2015, the N0 treatment slightly exceeded the N1 yield target of 14.5 Mg ha^−1^ (dry wt.), while the other 2015 treatments together averaged 22.1 Mg ha^−1^. Thus, the average 109 kg ha^−1^ of N mineralized in non‐manured soils during 2015 provided sufficient N even in plots with low preplant soil test N (Table 3). The uptake of N in harvested biomass given in kg ha^−1^ (i) decreased with silage yield and hence was smaller in 2016 relative to 2015, (ii) increased with initial soil FertN concentration, and (iii) increased with the addition of manure (Table 3). Manure may have increased N uptake in residual corn biomass by increasing root biomass and the root/shoot ratio relative to non‐manured soils (Lentz et al., 2014).

**TABLE 3:** Effect of treatments on corn silage yield, silage N, and silage C; results for class comparisons (contrasts); and least square means (*n* = 4).

	Silage yield		Silage N		Silage C	
	
Source of variation	*p* values					
Treatment	ns		*		ns	
Year	***		***		***	
Treatment × year	*		*		*	
Contrasts						
No‐Man vs. Man	ns		*		ns	
No‐FertN vs. FertN	**		**		**	
FertN linear (No‐Man)	*		*		ns	
FertN linear (Man)	ns		ns		ns	
Factor	Mg ha^−1^		kg ha^−1^		Mg ha^−1^	
Treatment	2015	2016	2015	2016	2015	2016
1	15.6b	12.4a	161.5b	130.7a	6.27a	5.28
2	21.6ab	12.9a	251.9a	146.7a	8.73a	5.53
3	20.8ab	14.4a	227.4a	179.2a	8.43a	6.09
4	20.8ab	9.4a	261.5a	114.6a	8.43a	4.01
5	21.8a	11.8a	280.2a	156.1a	8.87a	4.98
6	21.1a	12.8a	290.1a	162.5a	8.56a	5.45
Mean	20.3a	12.3b	245a	148b	8.21a	5.22b

*Note*: For each treatment within year, means followed by the same letter are not significantly different.

Abbreviations: FertN, organic‐N added; FertN linear, inorganic N levels are linearly related; Man, manure; No‐FertN, no inorganic‐N add‐ed; No‐Man, no‐manure; ns, not significant.

*, **, *** denote significance at 0.05, 0.01, and 0.001 probability levels, respectively.

### 3.5 Soil profile mobile N budget

Treatments strongly influenced the initial‐ and ending‐profile mobile‐N totals as well as the net mobile N loss (Table S7). Initial and ending profile total values increased both with increasing FertN and with the addition of manure, and FertN was positively related to these totals in both manured and non‐manured soils (Table 4; Table S7). In contrast, the effect of FertN on net mobile N loss differed when manure was added to soil. Net mobile N loss increased from −26.2 to 116 kg ha^−1^ in non‐manured soils in response to increasing FertN but decreased from −54.7 to −338 kg ha^−1^ in manured soils when FertN increased (Table 4). Thus, non‐manured soils amended with an average 318 kg N ha^−1^ in mineral fertilizer (N2) produced a positive net mobile N loss (116 kg ha^−1^), suggesting N leaching from the profile. On the other hand, adding an average 1388 kg N ha^−1^ in manure plus 318 kg N ha^−1^ in mineral fertilizer (N2) to the soil produced a negative net mobile N loss (−338 kg ha^−1^), suggesting net immobilization and adsorption of soil profile N during the year. These leaching results contrast with those reported for acidic loam soils, where N loss was (i) greater for manured soils relative to non‐manured (Frick et al., 2022) and (ii) did not increase linearly with N fertilizer amounts (Rupp et al., 2024).

**TABLE 4:** Mean mobile N loads, inputs, outputs, and net losses of mobile N in the soil profile (0–1.2 m) from late fall through summer's end for the six treatments.

	Treatment											
	1		2		3		4		5		6	
Initial + input mobile N												
N in soil WEOC (WEON*)	24.45	(2.09)	30.69	(3.70)	38.05	(2.88)	90.27	(10.22)	91.91	(7.98)	81.91	(4.82)
Soil inorganic N (InorgN*)	46.7	(7.0)	138.8	(14.7)	318.6	(24.5)	137.5	(20.8)	210.8	(24.4)	207.7	(20.0)
Cumulative net mineralized N	179.2	(21.8)	150.5	(23.7)	198.6	(26.7)	260.7	(23.7)	232.3	(18.2)	277.6	(40.9)
Total	250.3	(27.6)	319.9	(30.9)	555.2	(34.7)	488.5	(45.7)	535	(23.9)	567.2	(50.6)
Ending + output mobile N												
N in soil WEOC (WEON*)	9.44	(0.19)	12.93	(0.73)	13.59	(0.49)	25.6	(3.05)	23.72	(1.41)	24.11	(1.47)
Soil inorganic N (InorgN*)	88.1	(20.9)	101.8	(16.1)	176.7	(30.1)	260.5	(37.6)	429.8	(82.3)	571.6	(90.9)
N in harvested biomass	146.1	(10.8)	199.3	(24.4)	203.3	(20.4)	188.1	(29.9)	218.1	(26.4)	226.3	(25.7)
N in residual biomass	32.6	(2.4)	44.4	(5.4)	45.3	(4.5)	68.3	(10.9)	79.2	(9.6)	82.1	(9.3)
N lost in gas emissions	0.25	0.00	0.25	0.00	0.25	0.00	0.8	0.00	0.8	0.00	0.8	0.00
Total	276.6	(20.3)	358.8	(22.3)	439.1	(35.2)	543.2	(19.8)	751.6	(52.9)	905.0	(64.8)
Net mobile N loss	−26.2	(23.1)	−38.9	(26.0)	116	(47.1)	−54.7	(56.0)	−217	(51.2)	−338	(50.1)

*Note*: Values in parentheses are the standard error of the mean (*n* = 8). All values are given in kg ha^−1^.

Abbreviations: WEOC, water extractable organic carbon; WEON, water extractable organic nitrogen.

The manure's effect on net mobile N loss largely resulted from its disproportionate influences on initial‐ and ending‐profile mobile‐N totals. As a class, manure increased initial profile mobile‐N totals an average 1.4‐fold (530 vs. 375 kg ha^−1^) over that of non‐manure, yet it increased summer's‐end profile mobile‐N totals 2.1‐fold (733 vs. 358 kg ha^−1^) relative to non‐manure (Table 5). Part of the reason manure less effectively increased initial profile mobile‐N totals overall is because it inhibited the availability of InorgN added in the N2 FertN treatment. At the initial late fall sampling, the N2 FertN treatment that applied an average 318 kg N ha^−1^ had boosted non‐manured soil InorgN load to 319 kg ha^−1^, while it raised that of manured soils only to 208 kg ha^−1^ (Table S2). Al‐Saedi et al. (2021) reported that adding organic matter to calcareous soil increased NH_4_^+^ adsorption in the soil by 36%. In soils dominated by 2:1 type clay minerals (such as illite in the current study), up to 50% of NH_4_^+^ added in manure or fertilizer can be fixed by the clay; this slows nitrification of the NH_4_^+^, which is normally very rapid in these soils (Nieder et al., 2011; Sowden, 1976). Of the fertilizer NH_4_^+^ added to manured, calcareous soil and subsequently fixed in the interlayer of 2:1 type clays, >50% was released and became available to plants and microorganisms within 7–12 days (Green et al., 1994; Ranjbar & Jalali, 2014). Heterotrophic microorganisms can effectively incorporate NH_4_^+^ from the clay‐bound pool, favoring its release from mineral sites (Nieder et al., 2011). This temporary fixation and release of added NH_4_^+^ could inhibit its conversion to nitrate and reduce NO_3_^−^ losses from the soil system (Nieder et al., 2011).

**TABLE 5:** Mean mobile N loads, inputs, outputs, and net losses of mobile N in the soil profile (0–1.2 m) from late fall through summer's end for no‐manure and manure classes.

	Class	
	No manure (kg ha^−1^)	Manure (kg ha^−1^)
Initial + input mobile N		
N in soil WEOC (WEON*)	31b	88a
Soil inorganic N (InorgN*)	168	186
Cumulative net mineralized N	176b	257a
Total	375b	530a
Ending + output mobile N		
N in soil WEOC (WEON*)	12b	24a
Soil inorganic N (InorgN*)	122b	421a
N in harvested biomass	183b	211a
N in residual biomass	41b	77a
N lost in gas emissions	0	1
Total	358b	733a
Net mobile N loss	17a	−203b

*Note*: For each mobile N component, class means followed by the same letter are not significantly different. Letters are not displayed if the effect was not significant in the analysis of variance (ANOVA).

Abbreviations: WEOC, water extractable organic carbon; WEON, water extractable organic nitrogen.

At summer's end, manured soil profiles contained an average 3.5x more InorgN and 1.8x more WEOC than no‐manure soils. A relevant question then arises is will the mobile constituents in the manure‐treated soil profile leach from the soil root zone in the next year? Observations made in a separate study conducted on similar manure‐treated soils showed no increase in leaching of InorgN and WEOC from manured versus no‐manure soils the second year or even the third year after application (Lentz & Lehrsch, 2018). A long‐term study conducted on similar soils applied a one‐time, fall manure application (57–72 Mg ha^−1^, dry wt.) and compared net N mineralization over succeeding growing seasons in non‐manured, control, and mineral fertilized soils (Lentz & Lehrsch, 2012). The authors found that the manured soils continued to stimulate N‐cycling activity for up to 6 years post treatment, producing 1.9x greater net N mineralization on average than non‐manured soils during the six growing seasons following application (Table S8). This suggests that the stimulated N‐cycling in post‐treatment, manured soils may potentially inhibit InorgN and WEOC leaching for many years after application.

## 4 CONCLUSIONS

Experimental findings support the hypothesis that fall manure application to soil profiles containing excess InorgN can slow N translocation and loss through the profile and root zone. Evidence suggests that the delayed InorgN leaching results from its continual participation in successional immobilization–mineralization cycles, which are further stimulated by manure addition. It also appears that recurrent cycling of NH_4_‐N between the soil solution and exchangeable pools, such as absorption sites on added organic matter or fixation sites on the humic‐clay mineral complex, may further slow InorgN transport through manured soils.

Manure slightly increased the proportion of year‐long, cumulative net N mineralization in the 0.3‐ to 0.6‐m depth compared to the 0‐ to 0.3‐m depth, but the relationship was not a strong one. This implies that the leaching and subsequent mineralization of soluble organic N did not substantially contribute to N cycling in deeper soils. On the other hand, it may be that the cycling process was still active and ongoing at 0.3‐ to 0.6‐m depth, which may disguise the net impact in the short term.

Other research suggests that manure's inhibiting effect on soil N loss in these calcareous soils may extend beyond one growing season due to its long‐term stimulation of N‐cycling processes. A single manure application to these soils can continue to stimulate N‐cycling activity for up to 6 years post treatment. This suggests that annual or even alternate‐year manure applications to these soils and consequent accrual of C and N could overwhelm the soils capacity to efficiently cycle InorgN. Recent research on these soils indicated that cropping systems using annual manure applications can increase leaching of InorgN relative to no‐manure soils (A. B. Leytem, personal communication, March 31, 2025). This suggests that manure applications be separated in time in order to reduce soil N loss.

## AUTHOR CONTRIBUTIONS

**Rodrick D. Lentz**: Conceptualization; data curation; formal analysis; investigation; methodology; project administration; resources; supervision; visualization; writing—original draft. **Jim A. Ippolito**: Investigation; methodology; resources; supervision; writing—review and editing.

## CONFLICT OF INTEREST STATEMENT

The authors declare no conflicts of interest.

## Supporting information



Additional supporting information can be found online in the Supporting Information section at the end of this article.
