# Supplementary material for: Fall‐applied manure can conserve excess soil‐profile inorganic‐N for the subsequent cropping year
Source: J Environ Qual. 2025 May 13;54(5):1003–16. doi: 10.1002/jeq2.70040 (PMC12431947; doi:10.1002/jeq2.70040)
Supplement: Supplementary file 1 — Additional supporting information can be found online in the Supporting Information section at the end of this article. [file JEQ2-54-1003-s001.docx]

SUPPLEMENTAL MATERIAL

**Fall-Applied Manure Can Conserve Excess Soil-Profile Inorganic-N for the Subsequent Cropping Year**

Rodrick D. Lentz^1^ and Jim A. Ippolito^2*^

^1^ Northwest Irrigation and Soils Research Laboratory, USDA-ARS, 3793 N. 3600 E., Kimberly, ID 83341-5076, USA. (Retired)

^2^ School of Environment and Natural Resources, The Ohio State University, 2021 Coffey Rd, Columbus, OH 43210, USA.

^*^ Correspondence:

Jim A. Ippolito, School of Environment and Natural Resources, The Ohio State University, 2021 Coffey Rd, Columbus, OH 43210, USA. Email: ippolito.38@osu.edu

Accepted in *Journal of Environmental Quality* on 15 April 2025

**Supplemental Information (14 pages):**

**Climate and Soil Temperature Data**

**Tables S1 to S8**

**Figures S1 to S4**

**Climate and Soil Temperature Data**

The climate during the two years produced winter (Nov. to Feb.) air temperatures 1.7°C

above average in 2015 and 0.6°C below average in 2016 (**Fig. S1**). Spring (Mar-Apr) air temperatures were above average in both 2015 (1.6°C) and 2016 (4.2°C), while May to Sept temperatures were slightly above average, more so for 2015 than 2016. Near-surface soil temperatures generally tracked those of the air but were influenced by irrigation events and manured soils tended to be warmer than non-manured soils (**Fig. S2**).

**Table S1. Field operation and sampling events calendar**

|  |  | Experiment One |  | Experiment Two |  |
| --- | --- | --- | --- | --- | --- |
| Event |  | Date |  | Date |  |
| Soil profile baseline samples collected |  | 26 Nov 2014 |  | 14 Oct 2015 |  |
| Manure applied |  | 02 Dec 2014 |  | 12 Nov 2015 |  |
| Urea fertilizer applied |  | 11 Dec 2014 |  | 13 Nov 2015 |  |
| Soil disk plowed to 15-cm depth |  | 12 Dec 2014 |  | 16 Nov 2015 |  |
| Soil profile late fall samples collected |  | 12 Dec 2014 |  | 24 Nov 2015 |  |
| Winter buried bags placed |  | 17 Dec 2014 |  | 08 Dec 2015 |  |
| Winter buried bags removed |  | 30 Apr 2015 |  | 22 Apr 2016 |  |
| Soil profile Spring samples collected |  | 27 May 2015^H^ |  | 6 May 2016 |  |
| Corn planted (Round-up ready) |  | 13 May 2015 |  | 04 May 2016 |  |
| Spring, Early Summer, and Late Summer buried bags placed |  | 28 May 2015 |  | 10 May 2016 |  |
| Spring buried bag removed |  | 16 June 2015 |  | 15 June 2016 |  |
| Early Summer buried bag removed |  | 04 Aug 2015 |  | 03 Aug 2016 |  |
| Corn biomass and yield sampled |  | 10 Sept 2015 |  | 07 Sept 2016 |  |
| Late Summer buried bag removed |  | 16 Sept 2015 |  | 15 Sept 2016 |  |
| Soil profile Summer End samples collected |  | 15 Oct 2015 |  | 25 Oct 2016 |  |
| ^H^ Sampling delayed 11 days by rain | | | | |  |

**Table S2. Effect of FertN and manure treatments on 0- to 1.2-m soil inorganic N load (InorgN*) for each sampling date, results for class comparisons (contrasts), and least-square-mean values for treatments.**

|  | Late Fall | | Spring | | Summer’s end | |
| --- | --- | --- | --- | --- | --- | --- |
| **Source of Variation** | ----------------------------------- *P*-values ----------------------------------- | | | | | |
| Treatment | *** | | *** | | *** | |
| Year | *** | | *** | | *** | |
| Treatment×Year | ns | | ** | | *** | |
|  |  | |  | |  | |
| Contrasts ^†^ |  | |  | |  | |
| No-Man vs. Man | ns | | *** | | *** | |
| No-FertN vs FertN | *** | | *** | | *** | |
| FertN linear (No-Man) | *** | | *** | | ** | |
| FertN linear (Man) | ** | | *** | | *** | |
|  |  | |  | |  | |
| FertN treatment | No Manure | Manure | No Manure | Manure | No Manure | Manure |
|  | ------------------------------ kg ha^-1^ ------------------------------ | | | | | |
| N0 | 46.7 | 138 | 102 | 336 | 88.1 | 260 |
| N1 | 139 | 211 | 230 | 426 | 102 | 430 |
| N2 | 319 | 208 | 357 | 515 | 177 | 572 |
|  |  |  |  |  |  |  |
| Average | 168 | 186 | 230 | 426 | 122 | 421 |
|  |  | |  | |  | |
| * Significant at the 0.05 probability level.  ** Significant at the 0.01 probability level.  *** Significant at the 0.001 probability level.  † No-Man=No-Manure, Man=Manure, No-FertN=No inorganic-N added, FertN= Organic-N added, FertN linear=Inorganic N  levels are linearly related. | | | | | | |

**Table S3. FertN and manure treatment effects on soil Inorganic-N (InorgN) loads at late fall and summer’s end at each soil depth, class comparisons (contrasts), and treatment least square means (across two experiments).**

|  | --------------------------------------- Soil depth --------------------------------------- | | | | | | | | | | | |
| --- | --- | --- | --- | --- | --- | --- | --- | --- | --- | --- | --- | --- |
|  | 0.0 – 0.3 m | | 0.3 – 0.6 m | | | 0.6 – 0.9 m | | | 0.9 – 1.2 m | | | |
|  | **Late fall soil sampling** | | | | | | | | | | | |
| Source of Variation | --------------------------------------------------- *P*-values --------------------------------------------------- | | | | | | | | | | | |
| Treatment | *** | | | * | | | ns | | | ns  ns | | |
| Year | ** | | | ns | | | ns | | | ns | | |
| Treatment×Year | ns | | | ns | | | ns | | | ns | | |
| Contrasts ^†^ |  | | |  | | |  | | |  | | |
| No-Man vs. Man | ns | | | ns | | | ns | | | ns | | |
| No-FertN vs FertN | *** | | | ns | | | ns | | | ns | | |
| FertN linear (No-Man) | *** | | | ** | | | ns | | | ns | | |
| FertN linear (Man) | ** | | | ns | | | ns | | | ns | | |
|  |  | | | | | | | | | | | |
|  | --------------------------------------------- (kg ha^-1^) --------------------------------------------- | | | | | | | | | | | |
| Treatments ‡ | No Man | Manure | | No Man | Manure | | No Man | Manure | | No Man | | Manure |
| N0 | 32.8 | 116 | | 12.2 | 21.4 | | 14.6 | 18.3 | | 17.3 | | 23.5 |
| N1 | 121 | 193 | | 19.7 | 17.3 | | 16.2 | 18.9 | | 16.7 | | 19.8 |
| N2 | 279 | 191 | | 39.1 | 16.7 | | 17.7 | 21.4 | | 17.4 | | 22.3 |
| Average | 145 | 167 | | 23.5 | 18.5 | | 16.2 | 19.5 | | 17.1 | | 21.9 |
|  |  | | | | | | | | | | | |
|  | **Summer’s end soil sampling** | | | | | | | | | | | |
|  | --------------------------------------------------- *P*-values --------------------------------------------------- | | | | | | | | | | | |
| Treatment | ***  ns | | | *** | | | *** | | | **  ns | | |
| Year | ** | | | *** | | | *** | | | *** | | |
| Treatment×Year | * | | | ns | | | *** | | | *** | | |
| Contrasts ^†^ |  | | |  | | |  | | |  | | |
| No-Man vs. Man | *** | | | *** | | | *** | | | ** | | |
| No-FertN vs FertN | *** | | | ** | | | *** | | | * | | |
| FertN linear (No-Man) | ns | | | ns | | | *** | | | ns | | |
| FertN linear (Man) | ns | | | *** | | | ns | | | ns | | |
|  |  | | | | | | | | | | | |
|  | --------------------------------------------- (kg ha^-1^) --------------------------------------------- | | | | | | | | | | | |
| Treatments ‡ | No Man | Manure | | No Man | Manure | | No Man | Manure | | No Man | Manure | |
| N0 | 35.1 | 81.8 | | 20.3 | 66.9 | | 11.4 | 75.4 | | 21.4 | 36.4 | |
| N1 | 35.0 | 82.3 | | 23.0 | 101 | | 21.6 | 158 | | 22.2 | 88.7 | |
| N2 | 45.5 | 86.9 | | 50.3 | 200 | | 38.5 | 235 | | 42.3 | 48.9 | |
| Average | 38.5 b | 83.7 a | | 31.2 b | 122 a | | 23.8 b | 156 a | | 28.6 b | 58.0 a | |
| * Significant at the 0.05 probability level.  ** Significant at the 0.01 probability level.  *** Significant at the 0.001 probability level.  ^†^ No-Man=No-Manure, Man=Manure, No-FertN=No inorganic-N added, FertN= Organic-N added, FertN  linear=Inorganic N levels are linearly related.  ^‡^ For each treatment or class within year, means followed by the same letter are not significantly different.  Letters are not displayed if the effect was not significant in the ANOVA. | | | | | | | | | | | | |

**Table S4. FertN and manure treatment effects on profile water extractable organic carbon (WEOC) loads at late fall and summer’s end at each soil depth and entire soil profile (WEOC*), and results for class comparisons (contrasts).**

|  | --------------------------------------- Soil depth --------------------------------------- | | | | | |
| --- | --- | --- | --- | --- | --- | --- |
|  | 0 – 0.15 m | 0.15 – 0.3 m | 0.3 – 0.6 m | 0.6 – 0.9 m | 0.9 – 1.2 m | Total, 0 – 1.2 m |
|  |  |  |  |  |  | (WEOC*) |
| **Late fall soil sampling** | | | | | | |
| Source of Variation | --------------------------------------------------- *P*-values --------------------------------------------------- | | | | | |
| Treatment | ***  ** | ns | ns | ns | ns  ns | *** |
| Year | ns | ns | * | ns | ns | * |
| Treatment×Year | ns | ns | ns | ns | ns | ns |
|  |  |  |  |  |  |  |
| Contrasts ^†^ |  |  |  |  |  |  |
| No-Man vs. Man | *** | * | ns | ns | ns | *** |
| No-FertN vs FertN | ns | ns | ns | ns | ns | ns |
| FertN linear (No-Man) | ns | ns | ns | ns | ns | ns |
| FertN linear (Man) | ns | ns | ns | ns | ns | ns |
|  |  |  |  |  |  |  |
| *Class Comparisons* ‡ | --------------------------------------------- (kg ha^-1^) --------------------------------------------- | | | | | |
| No-Manure | 178 b | 151 b | 231 | 49 | 41.5 | 557 b |
| Manure | 889 a | 187 a | 287 | 50 | 39.2 | 1364 a |
|  |  | | | | | |
| **Summer’s end soil sampling** | | | | | | |
|  | --------------------------------------------------- *P*-values --------------------------------------------------- | | | | | |
| Treatment | ***  ** | ***  ns | * | * | ns  ns | *** |
| Year | ns | ns | ** | ns | ** | ns |
| Treatment×Year | ns | ns | ns | * | ns | ns |
|  |  |  |  |  |  |  |
| Contrasts ^†^ |  |  |  |  |  |  |
| No-Man vs. Man | *** | *** | ** | ** | nc | *** |
| No-FertN vs FertN | ns | ns | ns | ns | ns | ns |
| FertN linear (No-Man) | ns | ns | ns | ns | ns | ns |
| FertN linear (Man) | ns | ns | ns | ns | ns | ns |
|  |  |  |  |  |  |  |
| *Class Comparisons* ‡ | --------------------------------------------- (kg ha^-1^) --------------------------------------------- | | | | | |
| No-Manure | 34.8 b | 33.9 b | 59.1 b | 45.4 b | 41.5 | 215 b |
| Manure | 100 a | 85.2 a | 99.4 a | 52.3 a | 41.9 | 379 a |
|  |  |  |  |  |  |  |
|  |  |  |  |  |  |  |
| ** Significant at the 0.05 probability level.*  *** Significant at the 0.01 probability level.*  **** Significant at the 0.001 probability level.*  *^†^ No-Man=No-Manure, Man=Manure, No-FertN=No inorganic-N added, FertN= Organic-N added, FertN*  *linear=Inorganic N levels are linearly related.*  *^‡^ Class means for a given depth with different letters are not significantly different.* | | | | | | |

**Table S5. Effect of FertN and manure treatments on minN (net N mineralization) for individual soil layers, measurement intervals, and years, and results for class comparisons (contrasts).**

|  | **0 - 0.3 m** | | | | | | | | | | | **0.3 - 0.6 m** | | | | | | | | | | | |
| --- | --- | --- | --- | --- | --- | --- | --- | --- | --- | --- | --- | --- | --- | --- | --- | --- | --- | --- | --- | --- | --- | --- | --- |
| **Source of** |  | |  | | | Early | | | Late | | |  | | |  | | | Early | | | Late | | |
| **Variation** | Winter | | Spring | | | summer | | | summer | | | Winter | | | Spring | | | summer | | | summer | | |
|  | 2015 | 2016 | 2015 | 2016 | 2015 | | 2016 | 2015 | | 2016 | 2015 | | 2016 | 2015 | | 2016 | 2015 | | 2016 | 2015 | | 2016 |  |
|  | ------------------------------------------------------------- *P*-values ------------------------------------------------------------- | | | | | | | | | | | | | | | | | | | | | |  |
| Treatment | ns | ** | ** | ns | * | | ** | ns | | ns | ns | | ns | ns | | ns | ns | | ns | ns | | ns |  |
|  |  |  |  |  |  | |  |  | |  |  | |  |  | |  |  | |  |  | |  |  |
| Contrasts ^†^ |  |  |  |  |  | |  |  | |  |  | |  |  | |  |  | |  |  | |  |  |
| No-Man vs. Man | ns | ** | *** | * | ** | | ** | ns | | ns | ns | | ns | ns | | ns | ns | | ** | ns | | ns |  |
| No-FertN vs FertN | ns | ns | ns | ns | ns | | ns | ns | | ns | ns | | ns | ns | | ns | ns | | ns | ns | | ns |  |
| FertN linear (No-Man) | ns | * | ns | ns | ns | | ns | ns | | ns | * | | ns | ns | | ns | ns | | ns | ns | | ns |  |
| FertN linear (Man) | ns | ns | ns | ns | ns | | ns | ns | | * | ns | | ns | ns | | ns | ns | | ns | ns | | ns |  |
|  |  |  |  |  |  | |  |  | |  |  | |  |  | |  |  | |  |  | |  |  |
| * Significant at the 0.05 probability level.  ** Significant at the 0.01 probability level.  *** Significant at the 0.001 probability level.  ^†^ No-Man=No-Manure, Man=Manure, No-FertN=No inorganic-N added, FertN= Organic-N added, FertN linear=Inorganic N levels are  linearly related. | | | | | | | | | | | | | | | | | | | | | | | |

**Table S6.** **Effect of manure and soil depth on cumulative net N mineralization from late fall through summer’s end (CumMinN) averaged across years. Given also as a percentage of the total from both depths, CumMinN* (n=4).**

|  |  |  | CumMinN | |  |
| --- | --- | --- | --- | --- | --- |
| Source of Variation | | | -------- *P*-values ------ | |  |
|  | | |  | |  |
| Manure | | | * | |  |
| Depth | | | *** | |  |
| Manure×Depth | | | 0.26 | |  |
|  | | |  | |  |
|  | | | Depth | |  |
| Class | | | 0 - 0.3 m | 0.3 - 0.6 m |  |
|  | | | ---------- (kg ha^-1^) ---------- | |  |
| No Manure | | | 142.0 b | 34.1 c |  |
| Manure | | | 198 a | 58.8 c |  |
|  | | |  |  |  |
|  | | | Percent of 0- to 0.6-m soil | |  |
| No Manure | | | 80.6 a | 19.4 b |  |
| Manure | | | 77.1 a | 22.9 b |  |
| * Significant at the 0.05 probability level.  ** Significant at the 0.01 probability level.  *** Significant at the 0.001 probability level.  ^†^ Parameter class and depth means followed by the same letter are not  significantly different, | | | | |  |

**Table S7. Effect of FertN and manure treatments on 0- to 1.2-m, mobile (soluble) N soil budget components and results for class comparisons (contrasts) (n=4).**

|  | Initial + input mobile N | Ending + output mobile N | Net mobile N loss |
| --- | --- | --- | --- |
| **Source of Variation** | ----------------------------------- *P*-values ----------------------------------- | | |
| Treatment | *** | *** | *** |
| Year | *** | *** | ns |
| Treatment×Year | ns | *** | ** |
|  |  |  |  |
| Contrasts ^†^ |  |  |  |
| No-Man vs. Man | *** | *** | *** |
| No-FertN vs FertN | *** | *** | * |
| FertN linear (No-Man) | *** | *** | * |
| FertN linear (Man) | ns | *** | *** |
|  |  |  |  |
| * Significant at the 0.05 probability level.  ** Significant at the 0.01 probability level.  *** Significant at the 0.001 probability level.  † No-Man=No-Manure, Man=Manure, No-FertN=No inorganic-N added, FertN= Organic-N added, FertN linear=Inorganic N  levels are linearly related. | | | |

**Table S8. Cumulative net N mineralization (mid-April through mid-Sept.) in years following a one-time mean 69 Mg ha^-1^ (dry wt.) fall manure application.** ^†^

| No Manure | -------------------------- Years after manure application -------------------------- | | | | | |
| --- | --- | --- | --- | --- | --- | --- |
| Mean (all yrs) | 1 | 2 | 3 | 4 | 5 | 6 |
| ----------------------------------------------- mg N kg^-1^ soil ----------------------------------------------- | | | | | | |
| 19.1 c ^I^ | 51.3 a | 27.7 b | 33.6 b | 35.2 b | 25.5 b | 39.0 ab |
|  | | | | | | |
| ----------------------------------- Ratio (manure/no-Manure) ----------------------------------- | | | | | | |
| 1.0 | 2.7 | 1.5 | 1.8 | 1.8 | 1.3 | 2.0 |
| ^†^ Source: Lentz and Lehrsch (2012) and unpublished data derived from extended  years of observation from the same experiment.  ^I^ Least squared means followed by the same letter are not significantly different. | | | | | | |

***
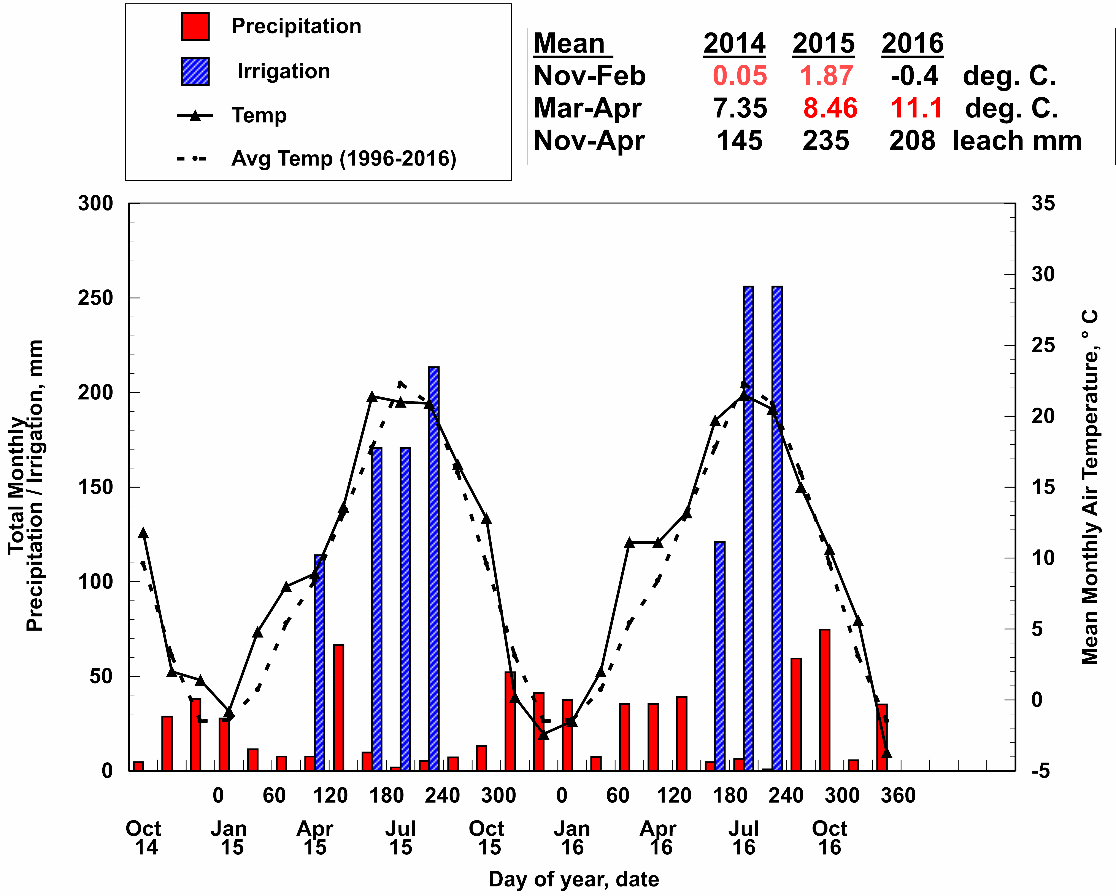
***

**Fig. S1**. Total monthly precipitation and irrigation inputs, mean monthly air temperature, and 1992-2016 average monthly air temperature at the study site from Oct. 2014 through Dec. 2016. The leach mm value is the sum of precipitation and irrigation amounts from Nov (previous yr) through Apr.


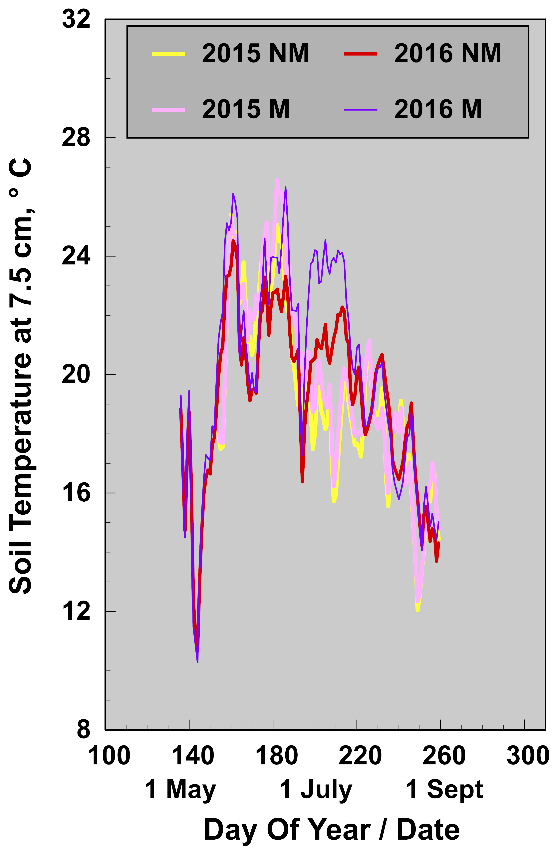


**Fig. S2**. Soil temperature at 7.5 cm depth in no manure (NM) and manured (M) plots during the 2015 and 2016 growing seasons.


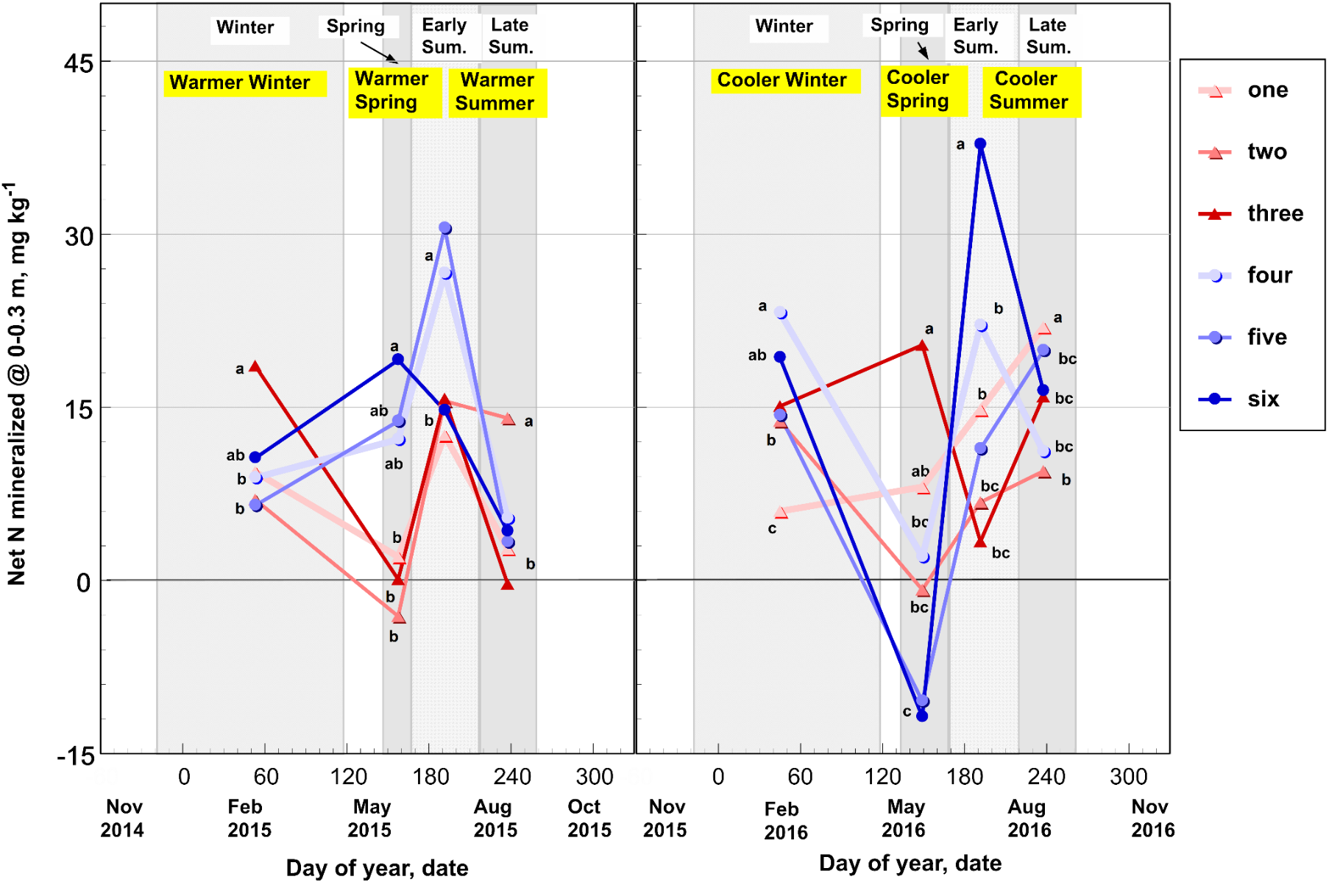


**Fig. S3**. The effect of inorganic N and manure amendments on net N mineralization at 0-to-0.3-m soil depth for defined periods during Exp. 1 and Exp. 2. Treatment means are significantly different if labeled with different lower case. Back panels in the figure identify the measurement interval used for each data point. Treatments #1-3 received no manure while #4-6 received manure in late fall, and treatments #1-3 and #4-6 received late fall, 10, 45, and 80 mg N kg^-1^ applied, respectively**
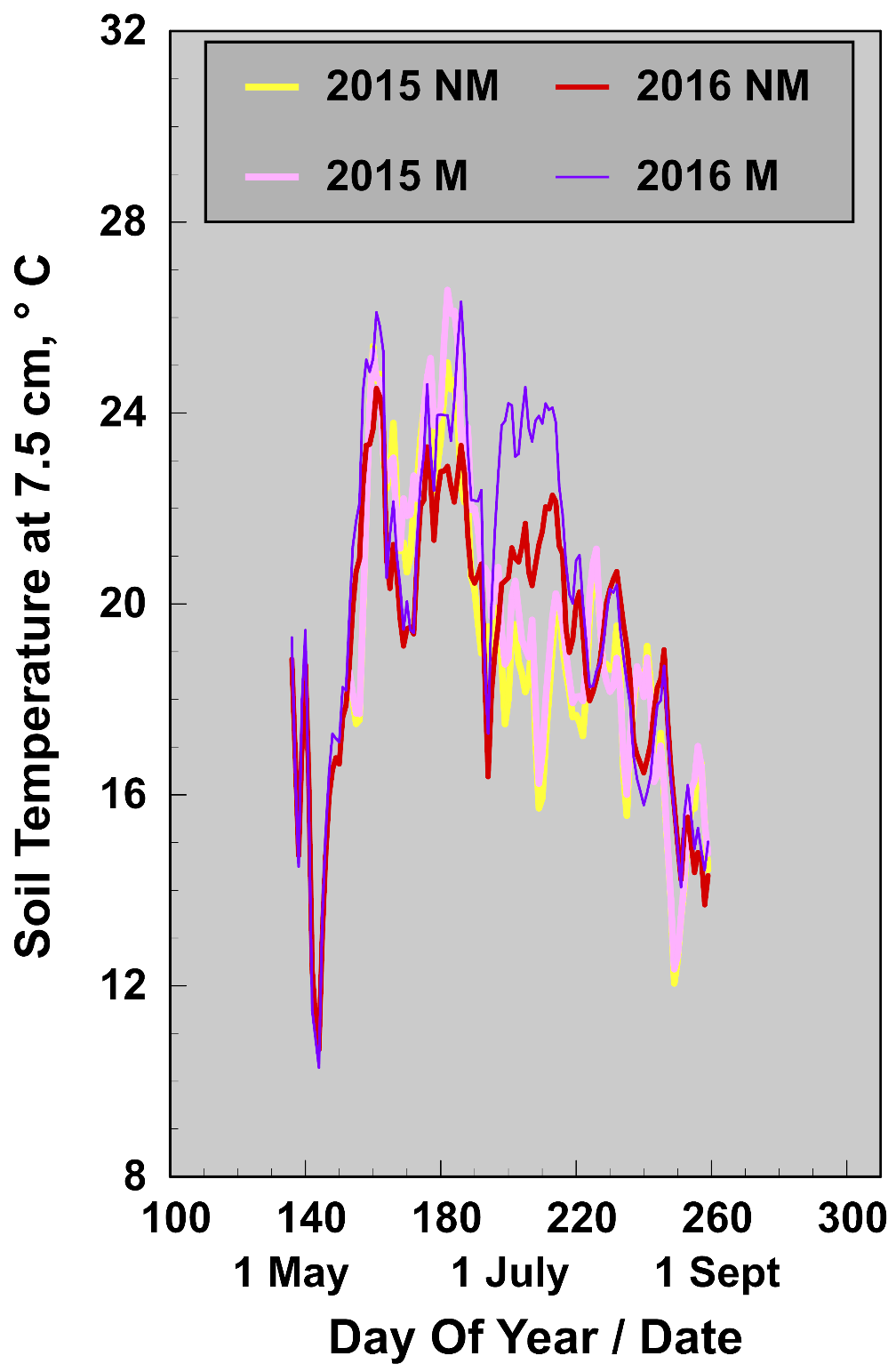
**.

**
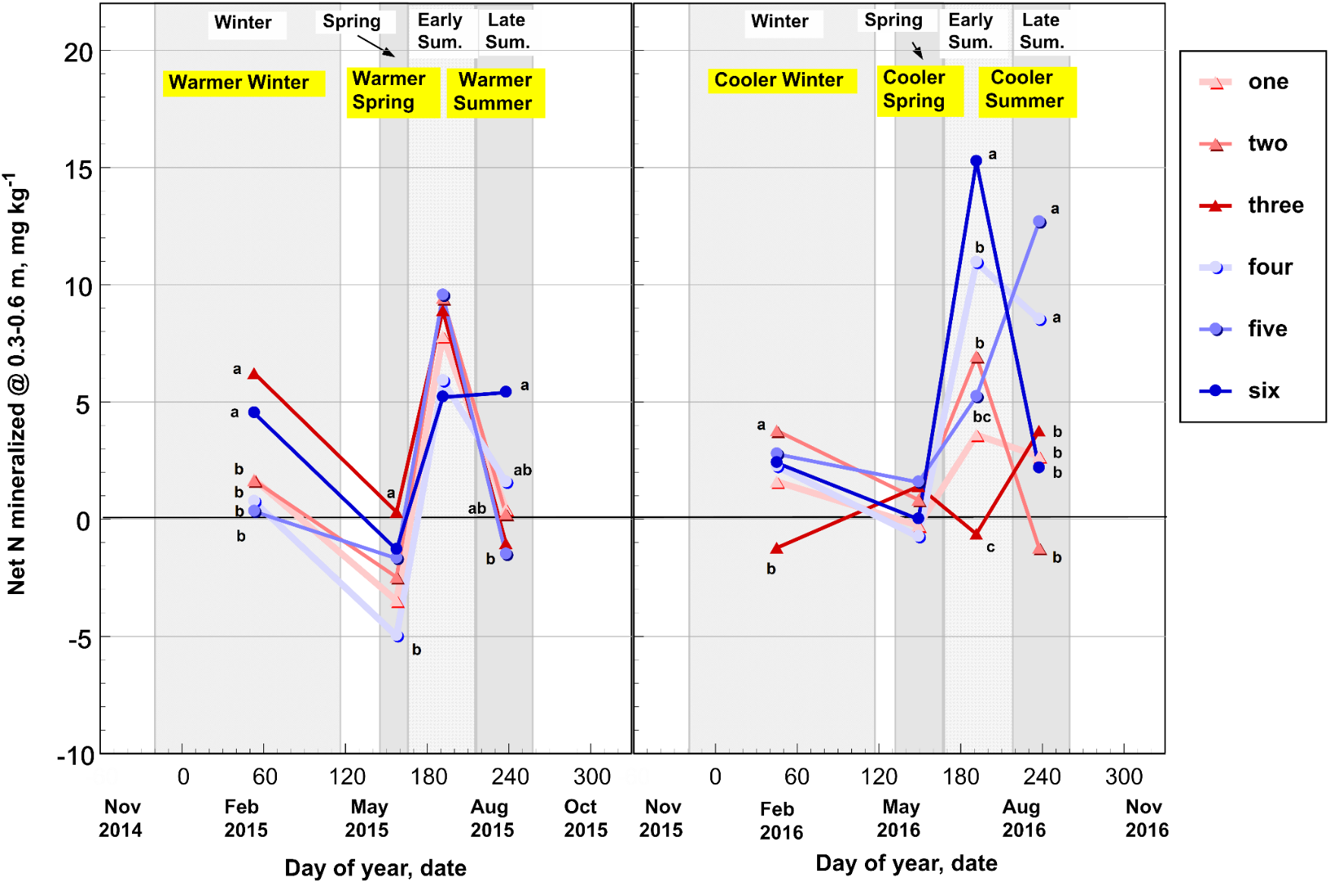
**

**
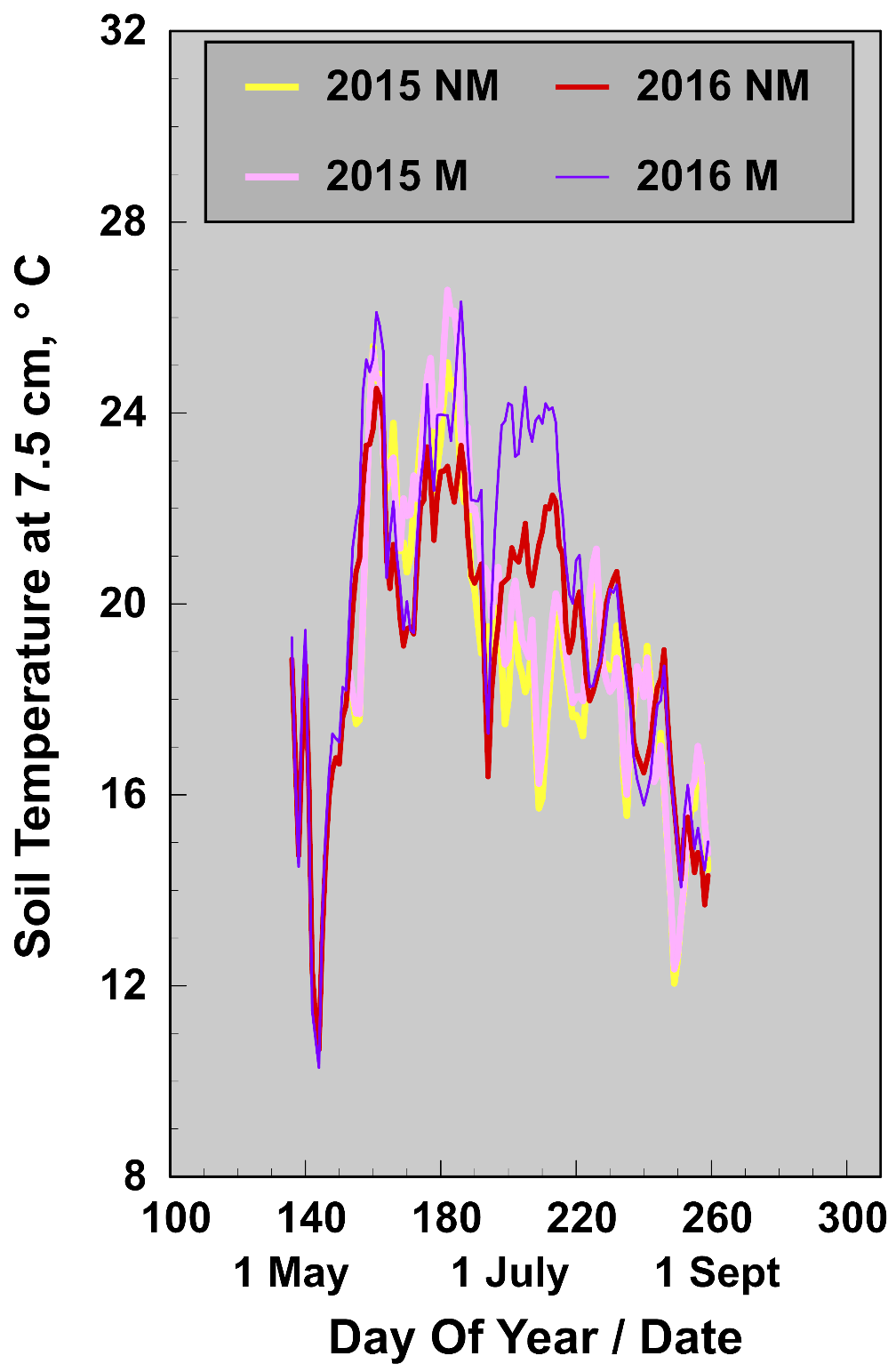
**

**Fig. S4.** The effect of inorganic N and manure amendments on net N mineralization at 0.3-to-0.6-m soil depth for defined periods during Exp. 1 and Exp. 2. Treatment means are significantly different if labeled with different lower case. Back panels in the figure identify the measurement interval used for each data point. Treatments #1-3 received no manure while #4-6 received manure in late fall, and treatments #1-3 and #4-6 received late fall, 10, 45, and 80 mg N kg-1 applied, respectively.
